# A Suppression Method for Random Errors of IFOG Based on the Decoupling of Colored Noise-Spectrum Information

**DOI:** 10.3390/mi16080963

**Published:** 2025-08-21

**Authors:** Zhe Liang, Zhili Zhang, Zhaofa Zhou, Hongcai Li, Junyang Zhao, Longjie Tian, Hui Duan

**Affiliations:** 1 Intelligent Control Laboratory, PLA Rocket Force University of Engineering, Xi’an 710025, China; 2 Institute of Optics and Electronics, School of Instrumentation Science and Optoelectronics, Engineering, Beihang University, Beijing 100191, China

**Keywords:** initial alignment, fiber-optic gyroscope, strapdown inertial navigation system, random error, time series model, spectral decoupling, adaptive Kalman filtering

## Abstract

In high-precision inertial navigation systems, suppressing the random errors of a fiber-optic gyroscope is of great importance. However, the traditional rule-based autoregressive moving average modeling method, when applied in Kalman filtering considering colored noise, presents inherent disadvantages in principle, including inaccurate state equations and difficulties in state dimension expansion. To this end, the noise characteristics in the fiber-optic gyroscope signal are first deeply analyzed, a random error model form is clarified, and a new model-order determination criterion is proposed to achieve the high-precision modeling of random errors. Then, based on the effective suppression of the angle random walk error of the fiber-optic gyroscope, and combined with the linear system equation of its colored noise, an adaptive Kalman filter based on noise-spectrum information decoupling is designed. This breaks through the principled limitations of traditional methods in suppressing colored noise and provides a scheme for modeling and suppressing fiber-optic gyroscope random errors under static conditions. Experimental results show that, compared with existing methods, the initial alignment accuracy of the proposed method based on 5 min data of fiber-strapdown inertial navigation is improved by an average of 48%.

## 1. Introduction

Fiber-optic gyroscopes (FOGs) serve as a core measuring component in a strapdown inertial navigation system (SINS), featuring significant advantages of an all-solid-state structure and a low cost. Among them, the interferometric fiber-optic gyroscope (IFOG) has reached a relatively mature stage of development and is currently the mainstream attitude-measuring element applied in a fiber-optic SINS. However, since it is restricted due to the working mechanism of IFOG, environmental factors such as temperature and vibration have a significant impact on its output signal, introducing measurement errors, namely gyro drift. This drift can be specifically divided into systematic drift and random drift. The former can be compensated through calibration, while the influence of the latter becomes the most important indicator for measuring the accuracy of IFOG. However, the influencing factors of IFOG errors do not come from a single source. Due to the combined effects of the device’s optical path, circuit, and external environment, the error term is superimposed with multiple noises with different correlation times, mainly including quantization noise (QN), angle random walk (ARW), bias instability (BI), rate random walk (RRW), and rate ramp (RR), etc. [1]. However, QN has been effectively improved with the sampling frequency of control hardware at present, and RR is more in line with the characteristics of trend-term errors. Therefore, the main factors causing the volatility of IFOG signals are ARW, BI, and RRW. Among them, ARW is angular-rate white noise, while BI and RRW belong to colored noise.

The accurate modeling and effective suppression of the random error terms in gyros are of great engineering significance. In this regard, scholars have carried out extensive research. A series of random error suppression methods for IFOG have been proposed, including low-pass filtering (LPF), wavelet transform (WT), empirical mode decomposition (EMD), Kalman filtering, etc. [2,3,4,5]. Among them, methods such as low-pass filtering, WT, and EMD are too complex for the limited computing resources of a SINS and difficult to implement, resulting in poor real-time performance of the algorithms [6]. As a time-domain filtering method, Kalman filtering is a recursive optimal estimation method, which offers incomparable advantages over other filtering algorithms in the field of inertial navigation with high real-time requirements and limited computing resources [7,8]. The autoregressive moving average (ARMA) model is a commonly used tool in time series analysis, and it is a combined form of the autoregressive model (AR) and the moving average model (MA). The three models can be used alone or in combination, according to the characteristics of the research object. Based on the characteristics of such models, Li Yang et al. [9] proposed a decoupled adaptive Kalman filtering method for IFOG signal denoising. The Allan variance analysis method was adopted to estimate the variance parameters of the measurement noise, and the time series analysis method was used to establish an AR (2) model for the random noise of IFOG. Although it effectively solves the problem of coupling between system noise variance and measurement noise variance in the adaptive Kalman filtering process, it weakens the weight of measurement error estimation in the adaptive filter, thereby limiting the filtering accuracy [9]. Yunpeng Tian et al. [10] conducted a time series analysis on the measured data of IFOG actually used in engineering, established an AR model for noise by using the recursive least square method, and introduced adaptive Kalman filtering processing. However, it only conducts separate analyses of various noises based on the analytical theory of Allan variance, without considering the coupling problem existing between low-frequency noises during the filtering process [10]. Lidong Wang et al. [11] adopted an improved AR (2) model for the random errors of IFOG and accelerometers in the FOG north seeker, and they established the random error models of FOG and accelerometers online. However, in the modeling process of random errors, only the empirical-based AR model-order determination method is adopted, which limits the effect of suppressing random errors of FOG [11]. Narasimhappa, M. et al. [12] proposed an adaptive robust Kalman filter based on the AR model to suppress the random noise of FOG, and they achieved good results, but they did not mention the modeling process of the AR model. Although it achieves a relatively ideal suppression effect on white noise, its suppression effect on colored noise is unsatisfactory [12]. Jin, S. et al. [13] proposed an adaptive Kalman filtering method based on the improved AR model, which achieved good filtering results, but did not provide the method for determining the optimal order of the model. Although the proposed improved AR model shows good mean-following performance under the condition of small-range data fluctuations, it still adopts a low-order model based on experience and does not clearly provide the basis for determining the optimal order of the model [13]. Song, N. et al. [14] proposed an adaptive Kalman filtering method based on the AR model, and they also did not focus on the discussion of the modeling process. Although it can improve the filtering effect to a certain extent, it only suppresses the amplitude of white noise and cannot effectively suppress colored noise [14]. The above methods have all played a certain role in suppressing the random walk noise of a FOG.

In addition to the filtering methods based on the AR model, some scholars have also proposed filtering methods based on the ARMA model, considering the influencing factors of colored noise. Zeng Qinghua et al. [15] addressed the issue of the ARMA model being unable to expand dimensions by using a high-order AR model to approximate the MA term, and they introduced the MA term based on the white noise estimate as a control term into the adaptive Kalman filter. Compared with the traditional AR model-based filtering method, this approach improved the noise variance suppression effect by more than 10%. However, in essence, it mainly suppresses the white noise of the FOG [15]. Xiong Xue et al. [16] proposed an improved Kalman filtering method based on colored noise, and they established a state prediction covariance formula for colored noise, but they approximated the colored noise as a first-order AR process [16]. Yang Bo et al. proposed an improved Kalman filtering algorithm under colored noise conditions, which also approximates the colored noise as a first-order Markov process [17]. In the adaptive filtering method for random noise of FOG considering colored noise, Jin Kaidi et al. [18] used the extended Harvey equation to realize the whitening of colored noise. Although this avoids the artificial approximation in the previous methods, the order of the ARMA model is determined by artificially specified criteria. As a result, the established ARMA model cannot accurately reflect the true distribution of white noise and colored noises in FOG [18].

To address the existing problems in the above-mentioned research, this paper first conducts an in-depth analysis and discussion on the noise composition characteristics in the IFOG signal, and clarifies the form of the IFOG random error model. Then, according to the composition characteristics of the noise, a new AR model-order determination criterion is proposed to achieve high-precision modeling of colored noise. Finally, based on the linear system model of colored noise, an adaptive Kalman filter is constructed to realize the real-time decoupling estimation of noise coefficients, which overcomes the principled defects of the traditional ARMA model and effectively suppresses the white noise and colored noise of the IFOG. The feasibility and effectiveness of the proposed method are verified through the random error suppression experiment of the IFOG and the initial alignment test of the strapdown inertial navigation system. Table 1 summarizes several advantages of the method proposed in this paper compared with the traditional rule-based ARMA modeling method.

## 2. Noise Composition Analysis and Improved High-Precision Modeling Scheme for IFOG

All IFOG data used in this paper were collected under static conditions, and the measured values are the zenith component of the Earth’s rotation angular velocity at the latitude (34°01′ North Latitude) of the experimental site (theoretically, its angular velocity output should be a constant, but due to the existence of random errors, the actual angular velocity output fluctuates irregularly over time, which generally conforms to a first-order Gaussian Markov process).

The noise sources of IFOG are complex, mainly including the following: light source noise represented by relative intensity noise, detection circuit noise represented by shot noise, and optical path transmission noise represented by the Rayleigh backscattering effect and the Kerr effect, as well as other noises such as optical path device noise, background noise, temperature gradient noise, and electronic thermal noise. If these errors are classified according to power spectral characteristics and time correlation, the high-frequency component of relative intensity noise, shot noise, Rayleigh backscattering, background noise, electronic thermal noise, etc. can be categorized into angular-rate white noise (i.e., angle random walk, ARW), while the Kerr effect, temperature gradient noise, the medium-low frequency component of relative intensity noise, optical path device noise, etc. fall into the category of angular rate colored noise (mainly bias instability, BI, or angular rate random walk, RRW) [19,20,21]. It can be seen from the above analysis that the most important factor affecting the stability of the IFOG signal is the ARW error, while the BI error and RRW error are also important influencing factors [22]. Next, the theoretical characteristics of these three main errors will be analyzed.

The power spectral density (PSD) is used to describe the ARW error of the IFOG, and its equation is

(1) $$S_{\mathrm{ARW}}(f)=N^{2}$$

In Equation (1), N is the ARW error coefficient. The PSD equation of the BI error is

(2) $$S_{BI}(f)=\left\{\begin{array}{l} I^{2}/(2\pi f),f\leq f_{0}\\ 0,f\geq f_{0} \end{array}\right.$$

In Equation (2), I is the BI error coefficient, and $f_{0}$ is the cutoff frequency. The PSD equation of the RRW error is

(3) $$S_{RRW}(f)={\left(\frac{K}{2\pi }\right)}^{2}\frac{1}{f^{2}}$$

In Equation (3), K is the RRW error coefficient.

Equations (1)–(3) have, respectively, described the power spectral characteristics of the three noises of the IFOG (ARW, BI, and RRW). According to the relationship between the power spectral function and the system transfer function [23]

(4) $$S_{\Omega }(j\omega )=|G(j\omega ){|}^{2}$$

the system transfer functions corresponding to the three noises can be derived. Among them, the system transfer function of the ARW error is

(5) $$G_{ARW}(j\omega )=N$$

the system transfer function of the BI error is

(6) $$G_{BI}(j\omega )=\frac{I}{\sqrt{j\omega }}\approx \frac{\beta I}{j\omega +\beta }$$

In Equation (6), β is the inverse correlation time constant, which is the reciprocal of the noise correlation time. Since the BI error generally exhibits low-frequency characteristics (0.01–10 Hz), the above approximation is performed to facilitate subsequent analysis. The system transfer function of the RRW error is

(7) $$G_{RRW}(j\omega )=\frac{K}{j\omega }$$

Thus, according to the linear system theory, the following models can be further used to describe these three kinds of noises

(8) $$Y_{ARW}(j\omega )=NX_{ARW}(j\omega )$$

(9) $$Y_{BI}(j\omega )=\frac{\beta I}{j\omega +\beta }X_{BI}(j\omega )$$

(10) $$Y_{RRW}(j\omega )=\frac{K}{j\omega }X_{RRW}(j\omega )$$

In Equations (8)–(10), $X_{ARW}(j\omega )$, $X_{BI}(j\omega )$, and $X_{RRW}(j\omega )$ are the Gaussian white noise inputs to the system, while $Y_{ARW}(j\omega )$, $Y_{BI}(j\omega )$, and $Y_{RRW}(j\omega )$ are the system outputs. Furthermore, the differential equations corresponding to Equations (8)–(10) in the time domain can be derived as follows:

(11) $$y_{ARW}(t)=Nu_{ARW}$$

(12) $${\dot{y}}_{BI}(t)=-\beta {\dot{y}}_{BI}(t)+\beta Iu_{BI}$$

(13) $${\dot{y}}_{RRW}(t)=Ku_{RRW}$$

In Equations (11)–(13), $u_{ARW}$, $u_{BI}$, and $u_{RRW}$ are mutually independent Gaussian white noises corresponding to the three items of noise, with a mean of 0 and a variance of 1. The discretized forms of Equations (11)–(13) are as follows.

(14) $$y_{ARW,k}=\frac{N}{\sqrt{\Delta T}}u_{ARW}$$

(15) $$y_{BI,k}=(1-\beta \Delta T)y_{BI,k-1}+\beta \sqrt{\Delta T}Iu_{BI}$$

(16) $$y_{RRW,k}=y_{RRW,k-1}+K\sqrt{\Delta T}u_{RRW}$$

In Equations (14)–(16), ΔT is the discretization step size, i.e., the sampling period, and k is the observation time (k>1).

Inertial navigation has high requirements for the real-time performance of the algorithm, so the Kalman filtering method based on the ARMA model is often used to suppress the random error of the IFOG. The ARMA model is a composite form of the AR model and the MA model, which can generally be used alone or in combination, according to actual needs, and these three models can also be transformed into each other [24]. The general form of the ARMA model is

(17) $$A\left(B^{-1}\right)x_{k}=C\left(B^{-1}\right)\varepsilon_{k}$$

In Equation (17), $x_{k}$ represents the random time series. Meanwhile, the following definitions exist.

(18) $$A\left(B^{-1}\right)=1-\sum_{i=1}^{p}a_{i}B^{-i},\;C\left(B^{-1}\right)=1+\sum_{i=1}^{q}c_{i}B^{-i}$$

In Equation (18), $a_{i}$ and $c_{i}$ are the coefficients of the AR model and the MA model, respectively. p and q are the orders of the AR model and the MA model, respectively. B is the delay operator (for example, $B^{-i}x_{k}=x_{k-i}$), and $\varepsilon_{k}$ is the white noise sequence. Among them, the expression form of the AR model is

(19) $$A\left(B^{-1}\right)x_{k}=\varepsilon_{k}$$

The traditional order-determination methods for ARMA models (including AR models and MA models) can be divided into two types. The first is to perform autocorrelation and partial autocorrelation analysis on time series data and determine the model type and order by judging the truncation and tailing conditions. However, this method involves strong subjectivity and will introduce artificial modeling errors [25]. The second is the order-determination method based on information criteria, commonly including the Akaike Information Criterion (AIC) and Bayesian Information Criterion (BIC). By traversing different order combinations and calculating information values, it realizes objective and automated order selection, avoiding the introduction of manual judgment errors. However, the model order determined via this method is a comprehensive result of balancing the goodness of fit and order complexity, and it cannot find the optimal model order for approximating the random errors of IFOGs [26]. Analysis can also indicate that, in addition to their respective shortcomings, the two traditional methods also have the same principled limitation, that is, they both perform permutations and combinations of multiple sub-models of different types, so as to establish a complete model that can best meet the pre-specified criteria. This leads to two problems: First, the random error model may change randomly (possibly being an AR model, an MA model, or an ARMA model), which fails to provide a consistent model format for the subsequent Kalman filtering process and thus drastically increases the design complexity of the filter. Second, the established model cannot truly reflect the error distribution characteristics of the IFOG. For example, both the AR terms and MA terms in the ARMA model simultaneously contain colored noise and white noise information. As a result, the Kalman filter based on the ARMA model cannot effectively separate different types of noise. Additionally, when the ARMA model is used as the system equation of the Kalman filter, if the order of the MA terms exceeds 1, there will be difficulties in state dimension expansion, which also limits the filtering accuracy of the Kalman filter.

As analyzed earlier, the static output sequence of the IFOG contains both time-correlated colored noise and independent white noise (ARW error). From Equations (15) and (16), it is known that the principal component-colored noises of the IFOG conform to the characteristics of a first-order Markov process. Combined with the structural characteristics of the AR model in Equation (19), theoretically, the AR model can approximate the time-correlated terms in the IFOG output sequence with infinite precision. However, a reasonable model order should be set; otherwise, overfitting or underfitting will occur.

Since the model parameters are estimated based on the measurement signal sequence of the FOG, the fitting residuals of the AR model contain two types of white noise simultaneously: the colored noise model error (which can be approximately understood as the driving white noise term $\varepsilon_{k}$ in Equation (19), serving as the system noise in the subsequent Kalman filtering process) and the ARW error of the IFOG (which serves as the measurement noise in the subsequent Kalman filtering process). In all subsequent formula derivations, the symbol ‘^’ is defined as the flag for the numerical estimate of the corresponding variable.

It is assumed here that the colored noise model error $\varepsilon_{k}$ and the ARW error of the IFOG are independent of each other. According to the variance summation formula, the following relationship can be further derived.

(20) $$\sigma_{Mod}^{2}=\sigma_{\varepsilon }^{2}+{\hat{\sigma }}^{2}(\tau_{0})$$

Among them, $\sigma_{Mod}^{2}$ is the variance of the model fitting residuals, $\sigma_{\varepsilon }^{2}$ is the variance of the colored noise model error $\varepsilon_{k}$, and ${\hat{\sigma }}^{2}(\tau_{0})$ is the Allan variance corresponding to the ARW error under the sampling period ΔT [26,27]. According to Equation (20), the following model-order discriminant index can be defined.

(21) $$\eta =\frac{{\hat{\sigma }}^{2}(\tau_{0})}{\sigma_{Mod}^{2}}=\frac{{\hat{\sigma }}^{2}(\tau_{0})}{\sigma_{\varepsilon }^{2}+{\hat{\sigma }}^{2}(\tau_{0})},\;0<\eta \leq 1$$

It is easy to analyze that, when the value of the index η is closer to 1, it indicates that the accuracy of the AR model is higher. Therefore, modeling based on the constraint criterion of Equation (21) can avoid the occurrence of overfitting or underfitting, so as to establish a high-precision pure AR model for random errors.

In the modeling process of the method proposed in this paper, the prior information related to noise amplitude required mainly includes the white noise variance information of the IFOG output sequence (i.e., the variance of angle random walk error, whose amplitude is much higher than that of other noises and can be obtained by calculating the Allan variance). In the newly proposed model-order determination process of this paper, it is necessary to perform a series of proportional discrimination and matching between the root mean square error information of the IFOG output sequence fitted by the AR model and the white noise variance information, so as to determine the optimal order of the AR model (the autoregressive terms of the AR model can reflect the superimposed amplitude information of multiple colored noises in the IFOG). The modeling approach of the pure AR model proposed in this paper is equivalent to replacing the MA terms in the ARMA model with a high-order AR model, which is feasible based on the theoretical foundation of time series analysis [25]. Meanwhile, the pure AR model can clearly distinguish between white noise and colored noise in the IFOG’s random errors in its expression form, which significantly reduces the design complexity of the subsequent Kalman filter.

## 3. Noise-Spectrum Information Decoupling Method Based on Two Types of Models

As discussed earlier, when the traditional ARMA model is used as the system state equation, it suffers from inaccurate noise representation and difficulties in state dimension expansion during the Kalman filtering process. However, Equations (15) and (16) are the standard state equation formats for designing Kalman filters. If these two formulas are introduced as the system state equations in the Kalman filter to suppress colored noise, the two principled defects of the traditional ARMA model mentioned above can be effectively avoided. Since the output of the IFOG is a single-channel signal, all time-correlated errors are coupled together. Therefore, the effective decoupling and estimation of the PSD coefficients for the two noises, BI and RRW, are important prerequisites for the filtering approach proposed in this paper.

It can be analyzed that the random signals of the IFOG can be uniformly fitted and modeled based on Equation (19), while Equations (14)–(16) represent the physical models corresponding to each single noise component in the random signals. Therefore, the following relationships exist between the AR model of IFOG random errors established based on Equation (19) and Equations (14)–(16).

(22) $$\begin{array}{l} x_{M\mathrm{ea},k}=\sum_{i=1}^{p}a_{i}B^{-i}x_{M\mathrm{ea},k-i}+\varepsilon_{Mod,k}=y_{ARW,k}+y_{BI,k}+y_{RRW,k}\\ =(1-\beta \Delta T)y_{BI,k-1}+y_{RRW,k-1}+\beta \sqrt{\Delta T}Bu_{BI}+K\sqrt{\Delta T}u_{RRW}+\frac{N}{\sqrt{\Delta T}}u_{ARW} \end{array}$$

In Equation (22), $x_{M\mathrm{ea},k}$ is the time series of the IFOG measurement signal, and $\varepsilon_{Mod,k}$ is the fitting residual of the AR model, with the following relationships.

$$E[\varepsilon_{Mod,k}]=E[\frac{N}{\sqrt{\Delta T}}u_{ARW}]+E[\varepsilon_{k}],Var[\varepsilon_{Mod,k}]=Var[\frac{N}{\sqrt{\Delta T}}u_{ARW}]+Var[\varepsilon_{k}]$$

Since the ARW error belongs to the measurement noise in the Kalman filtering process, when only considering the suppression of the IFOG’s ARW error, the AR model of the colored noise part can be used as the system state equation, and Equation (22) can be simplified to the following Equation:

(23) $$\begin{array}{l} x_{k}=\sum_{i=1}^{p}a_{i}B^{-i}x_{k-i}+\varepsilon_{k}=y_{BI,k}+y_{RRW,k}\\ =(1-\beta \Delta T)y_{BI,k-1}+y_{RRW,k-1}+\beta \sqrt{\Delta T}Bu_{BI}+K\sqrt{\Delta T}u_{RRW} \end{array}$$

It is known that the white noises $\beta \sqrt{\Delta T}Bu_{BI}$ and $K\sqrt{\Delta T}u_{RRW}$ corresponding to the observation time k are both small quantities, so Equation (23) can be rewritten in the following approximate form:

(24) $$x_{k}\approx (1-\beta \Delta T)y_{BI,k-1}+y_{RRW,k-1}$$

Furthermore, it can be derived from Equation (24) that

(25) $$y_{BI,k-1}\approx \frac{x_{k-1}-x_{k}}{\beta \Delta T}$$

Equation (25) is the relationship between the random process $Y_{BI,k}=[y_{BI,1},y_{BI,2},y_{BI,3},\cdots ,y_{BI,k}]$ corresponding to the BI noise and the random process $X_{k}=[x_{1},x_{2},x_{3},\cdots ,x_{k}]$ corresponding to the AR model. By referring to the calculation method of the recursive Allan variance, the recursive variance of the random process $Y_{BI,k}$ can be calculated as follows.

(26) $${\hat{\sigma }}_{BI,k}^{2}=(1-\frac{1}{k-1}){\hat{\sigma }}_{BI,k-1}^{2}+\frac{1}{2(k-1)}{\left(y_{BI,k}-y_{BI,k-1}\right)}^{2}$$

In Equation (26), ${\hat{\sigma }}_{BI,k}^{2}$ is the variance corresponding to $Y_{BI,k}$, where ${\hat{\sigma }}_{BI,0}^{2}=0$. Similarly, the recursive variance of the random process $X_{k}$ can be obtained as follows.

(27) $${\hat{\sigma }}_{x,k}^{2}=(1-\frac{1}{k-1}){\hat{\sigma }}_{x,k-1}^{2}+\frac{1}{2(k-1)}{\left(x_{k}-x_{k-1}\right)}^{2}$$

In Equation (27), ${\hat{\sigma }}_{x,k}^{2}$ is the variance corresponding to $X_{k}$, where ${\hat{\sigma }}_{x,0}^{2}=0$. Based on Equations (24), (26), and (27), and according to the variance combination formula for random processes, the following relationship can be derived.

(28) $${\hat{\sigma }}_{x,k}^{2}=\frac{\left(k-1)({\hat{\sigma }}_{BI,k}^{2}+{\hat{\sigma }}_{RRW,k}^{2})+0.5k(m_{BI}^{2}+m_{RRW}^{2}\right)}{2k-1}$$

In Equation (28), ${\hat{\sigma }}_{RRW,k}^{2}$ is the variance of the random process $Y_{RRW,k}=[y_{RRW,1},y_{RRW,2},\cdots ,y_{RRW,k}]$. $m_{BI}$ and $m_{RRW}$ are the means of $Y_{BI,k}$ and $Y_{RRW,k}$, respectively. It can be further derived that

(29) $${\hat{\sigma }}_{RRW,k}^{2}=\frac{\left(2k-1){\hat{\sigma }}_{x,k}^{2}-0.5k(m_{BI}^{2}+m_{RRW}^{2}\right)}{k-1}-{\hat{\sigma }}_{BI,k}^{2}$$

According to Equations (26) and (29), the variances of $Y_{BI,k}$ and $Y_{RRW,k}$ can be calculated, respectively. Furthermore, based on the relationship between the variance of the random process and the variance of the driving white noise, the PSD coefficients of the BI noise and the RRW noise can be derived as

(30) $$I_{k}={\hat{\sigma }}_{BI,k}\sqrt{\frac{2-\beta }{\beta \Delta T}}={\hat{\sigma }}_{BI,k}\sqrt{\frac{2\Gamma_{BI}-1}{\Delta T}}$$

(31) $$K_{k}=\frac{{\hat{\sigma }}_{RRW,k}}{\Gamma_{RRW}}\sqrt{\frac{2\Gamma_{RRW}-1}{\Delta T}}$$

In Equation (30), $I_{k}$ is the estimated value of the PSD coefficient of the BI noise at time k, and $\Gamma_{BI}$ is the correlation time of the BI noise (0.1 s to 10 s) [28]. In Equation (31), $K_{k}$ is the estimated value of the PSD coefficient of the rate random walk noise at time k, and $\Gamma_{RRW}$ is the correlation time of the rate random walk noise (theoretically infinite, with an order of magnitude ≥ 10^4^ s in practical engineering).

Thus, by analyzing and deriving the relationship between the AR model and the noise physical model, the decoupling and estimation of the PSD coefficients of the two main types of colored noises are realized, providing effective and accurate prior information for the subsequent design of the Kalman filter based on the physical model.

## 4. Design of Adaptive Kalman Filter Considering Colored Noise Suppression

As discussed earlier, the AR model established based on the order-determination criterion proposed in this paper can achieve high-precision fitting of the time-correlated noise (i.e., colored noise) of the IFOG, and can further combine with the linear system equation to decouple and estimate the noise coefficients. However, in addition to the system-colored noise, the measurement data of the IFOG is also superimposed with measurement noise dominated by ARW noise. If this model predicts the system-colored noise based on the measurement data, it will lead to the distortion of the predicted colored noise information. Therefore, before the PSD coefficients of colored noise are accurately decoupled and estimated, it is necessary to estimate the accurate prediction values of the AR model. At this time, the AR model can be considered as the system state equation, and the ARW error can be taken as the measurement noise. An improved adaptive Kalman filter (denoted as KF-A) [29] is adopted to suppress the ARW error of the IFOG. The discrete state-space model of the KF-A filter is as follows.

(32) $$\left\{\begin{array}{l} L_{k}=\Phi_{k,k-1}L_{k-1}+\Gamma_{k}W_{k}\\ Z_{k}=H_{k}L_{k}+V_{k} \end{array}\right.$$

In Equation (32), $L_{k}$ is the state vector, $\Phi_{k,k-1}$ is the state transition matrix, $W_{k}$ is the system noise vector, $\Gamma_{k}$ is the system noise coefficient matrix, $Z_{k}$ is the observation value of the IFOG output signal, $H_{k}$ is the measurement matrix, and $V_{k}$ is the measurement noise. The specific definitions of each symbol are as follows.

$$\left\{\begin{array}{l} L_{k}={\left[x_{k},x_{k-1},x_{k-2},\ldots ,x_{k-p+1}\right]}^{T}\\ \Phi_{k,k-1}={\left[\begin{array}{ccccc} a_{1} & a_{2} & a_{3} & \cdots & a_{p}\\ 1 & 0 & 0 & \cdots & 0\\ 0 & 1 & 0 & \ddots & 0\\ \vdots & \ddots & \ddots & \ddots & \vdots \\ 0 & 0 & 0 & 1 & 0 \end{array}\right]}_{p\times p}\\ W_{k}={\left[\varepsilon_{k},0,\cdots ,0\right]}_{1\times p}^{T}\\ \Gamma_{k}={\left[\begin{array}{ccccc} 1 & 0 & 0 & \cdots & 0\\ 0 & 0 & 0 & \cdots & 0\\ 0 & 0 & 0 & \ddots & 0\\ \vdots & \ddots & \ddots & \ddots & \vdots \\ 0 & 0 & 0 & 0 & 0 \end{array}\right]}_{p\times p},\;\;H_{k}={\left[1,0,\cdots ,0\right]}_{1\times p},\;\;V_{k}=\frac{N}{\sqrt{\Delta T}}u_{ARW} \end{array}\right.$$

The statistical characteristics of $W_{k}$ and $V_{k}$ are as follows.

$$E[W_{k}]=0,E[V_{k}]=0,E[W_{k}{W_{k}}^{T}]=Q_{k},E[V_{k}V_{k}^{T}]=R_{k},E[W_{k}V_{k}^{T}]=0$$

Among them, $Q_{k}$ is the system noise variance at time k, and $R_{k}$ is the measurement noise variance at time k (i.e., ${\hat{\sigma }}^{2}(\tau_{0})$). The filtering process of KF-A is as follows, with the improved links in bold.


**Step No.**

**Filtering Steps**

**The Equations Corresponding to the Filtering Steps**
aSet initial value based on the training data

$L_{1},P_{1},r_{1},R_{1},q_{1},Q_{1},\Gamma_{1}$

bCalculation of weighting factors

$d_{k}=(1-b)/(1-b^{k+1})$

cOne-step forecast

${\hat{L}}_{k,k-1}=\Phi_{k,k-1}{\hat{L}}_{k-1}+{\hat{q}}_{k-1}$

dOne-step mean square error estimation

$P_{k,k-1}=\Phi_{k,k-1}P_{k-1}\Phi_{k,k-1}^{T}+{\hat{Q}}_{k-1}$

eFilter residual variance estimation

${\hat{r}}_{k}=(1-d_{k}){\hat{r}}_{k-1}+d_{k}(Z_{k}-H_{k}{\hat{L}}_{k,k-1})$



$\nu_{k}=Z_{k}-H_{k}{\hat{L}}_{k,k-1}-{\hat{r}}_{k}$


**f**

**Measurement noise estimation**


${\hat{R}}_{k}=(1-\frac{1}{k-1}){\hat{R}}_{k-1}+\frac{1}{2(k-1)}{\left(Z_{k}-Z_{k-1}\right)}^{2}$

gCalculating filter gain

$K_{k}=P_{k,k-1}{H_{k}}^{T}{\left[H_{k}P_{k,k-1}{H_{k}}^{T}+{\hat{R}}_{k}\right]}^{-1}$

hState estimation

${\hat{L}}_{k}={\hat{L}}_{k,k-1}+K_{k}[Z_{k}-H_{k}{\hat{L}}_{k,k-1}]$

iMean square error estimation

$P_{k}=[I-K_{k}H_{k}]P_{k,k-1}$


**j**

**Model accuracy discrimination**


${\hat{R}}_{k}\leq \eta [H_{k}P_{k,k-1}H_{k}^{T}+{\hat{R}}_{k}]$


**k**

**Variance matching**


$\nu_{k}v_{k}^{T}>H_{k}P_{k,k-1}H_{k}^{T}+{\hat{R}}_{k}$

lSystem noise estimate

${\hat{q}}_{k}=(1-d_{k}){\hat{q}}_{k-1}+d_{k}\left({\hat{X}}_{k}-\Phi_{k,k-1}{\hat{X}}_{k-1}\right)$



${\hat{Q}}_{k}=(1-d_{k}){\hat{Q}}_{k-1}+d_{k}(K_{k}\nu_{k}\nu_{k}^{T}K_{k}^{T}+P_{k}-\Phi_{k,k-1}P_{k-1}\Phi_{k,k-1}^{T})$



The subscript ‘_*k*,*k*−1_’ represents the one-step prediction value of the corresponding variable in the Kalman filter. P is the mean square error matrix, K is the filter gain matrix, q is the mean of the system noise, r is the mean of the measurement noise, $d_{k}=(1-b)/(1-b^{k+1})$ is the weight coefficient of the adaptive filter, b whose value range is generally [0.95,0.99], $\nu_{k}$ is the filtering residual at time k, and the definitions of other variables are consistent with the previous context.

The above KF-A filter can effectively suppress the ARW error while accurately estimating the predicted value ${\hat{x}}_{k}$ of the colored noise AR model. Further combining with the relevant recursive formulas in Section 3, it is possible to real-time decouple and estimate the PSD coefficients ${\hat{I}}_{k}$ and ${\hat{K}}_{k}$ of the IFOG’s BI noise and RRW noise. Next, based on the estimated prior information, a Kalman filter for suppressing colored noise (denoted as KF-B) can be constructed. The discrete state-space model of the KF-B filter is as follows.

(33) $$\left\{\begin{array}{l} L_{color,k}=\Phi_{color,k,k-1}L_{color,k-1}+\Gamma_{color,k}W_{color,k}\\ Z_{color,k}=H_{color,k}L_{color,k}+V_{color,k} \end{array}\right.$$

In Equation (33), $L_{color,k}$ is the state vector, $\Phi_{color,k,k-1}$ is the state transition matrix, $W_{color,k}$ is the system noise vector, $\Gamma_{color,k}$ is the system noise coefficient matrix, $Z_{color,k}$ is the observation value of the IFOG output signal, $H_{color,k}$ is the measurement matrix, and $V_{color,k}$ is the measurement noise. The specific definitions of each symbol are as follows.

$$\left\{\begin{array}{l} L_{color,k}={\left[{\hat{x}}_{k},y_{BI,k},y_{RRW,k}\right]}^{T}\\ \Phi_{color,k,k-1}=\left[\begin{array}{ccc} 0 & \left(1-\beta \Delta T\right) & 1\\ 0 & \left(1-\beta \Delta T\right) & 0\\ 0 & 0 & 1 \end{array}\right]\\ W_{color,k}={\left[u_{BI},u_{RRW}\right]}^{T}\\ \Gamma_{color,k}=\left[\begin{array}{cc} 1 & 1\\ \beta \sqrt{\Delta T}{\hat{I}}_{k} & 0\\ 0 & {\hat{K}}_{k}\sqrt{\Delta T} \end{array}\right],\;\;H_{color,k}=[1,0,0],\;\;V_{color,k}=\frac{N}{\sqrt{\Delta T}}u_{ARW} \end{array}\right.$$

**Figure 1:**
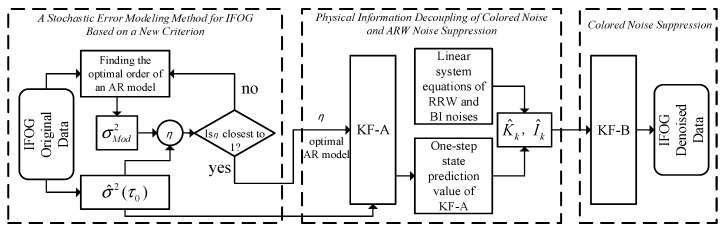
The process of modeling and suppressing random errors of IFOG using the method proposed in this paper.

The above is the design process of the adaptive Kalman filter, considering colored noise. From the derivation process, it can be intuitively seen that the filter is based on the physical equations and spectral information of the IFOG noise. In principle, it avoids the problems of inaccurate noise expression and difficult state dimension expansion existing in the Kalman filter based on the ARMA fitting model, thereby further improving the suppression effect on the random errors of the IFOG. The process of modeling and suppressing the random errors of IFOG by the method proposed in this paper is shown in Figure 1.

## 5. Simulation and Experiment

**Figure 2:**
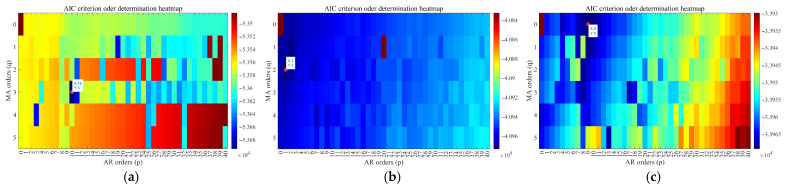
Heat map of AIC values three groups of IFOG simulation data. (**a**) The first group; (**b**) The second group; (**c**) The third group.

To verify the correctness of the analysis conclusions on different modeling schemes in Section 2 of this paper, three groups of simulation experiments were carried out. Three groups of IFOG simulation data with a duration of 5 h (denoted as total simulation data of IFOG) were generated by means of “first-order Gaussian Markov process” simulation. Among them, Gaussian white noise represents the measurement noise of IFOG, the first-order Markov process represents the time-correlated IFOG output signal that cannot be directly measured in practice, and the relevant parameters of Gaussian white noise and the first-order Markov process are set as shown in Table 2. Further, the first 30 min of simulation data in each group were used as training data. The ARMA model based on the AIC criterion and the pure AR modeling approach proposed in this paper were, respectively, adopted to fit the IFOG simulation data. The information of the two models obtained based on the three groups of training data is shown in Table 3. Figure 2 shows the heat map of AIC values during the order-determination process of the ARMA model, where the minimum AIC value (marked with a red circle in the figure) corresponds to the optimal order of the ARMA model.

**Figure 3:**
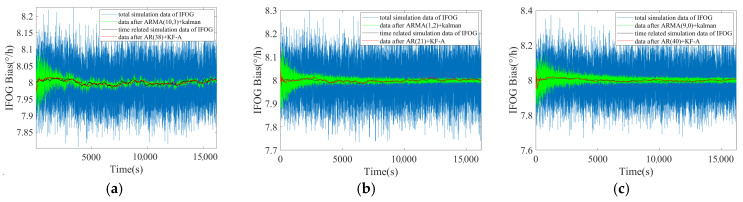
Filtering results of three groups of IFOG simulation data. (**a**) The first group; (**b**) the second group; (**c**) the third group.

Substitute the established ARMA model into the improved adaptive Kalman filter proposed in Reference [18] (denoted as ARMA (p, q) + Kalman), and substitute the established high-order AR model into the KF-A filter proposed in this paper (denoted as AR (p) + KF-A). Compare the differences between the filtering results of the two methods and the IFOG time-correlated term simulation data, so as to analyze the accuracy of different modeling methods in expressing the components of IFOG data. The filtering results of the two methods are shown in Figure 3. The blue line represents the IFOG simulation data containing measurement noise, the black line represents the simulation data of the time-correlated term of the IFOG output, the green line represents the filtering result of the “ARMA (p, q) + Kalman” method, and the red line represents the filtering result of the “AR (p) + KF-A” method. It can be intuitively seen from Figure 3a–c that both filtering methods can exhibit good suppression effects on random errors. Among them, the red line can maintain a high degree of consistency with the trend of the black line and show the characteristics of low-frequency fluctuations. However, compared with the blue line, the green line only reduces the overall amplitude, but still retains obvious high-frequency random errors.

**Figure 4:**
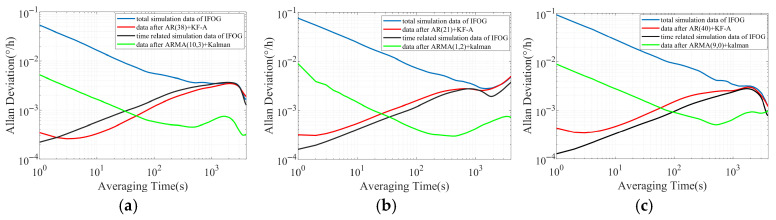
The double-logarithmic curves of Allan standard deviation for three groups of IFOG simulation data before and after filtering. (**a**) The first group; (**b**) The second group; (**c**) The third group.

Further, the Allan standard deviation double-logarithmic curves corresponding to all data in Figure 3 are plotted as shown in Figure 4. It can be seen from Figure 4a–c that the red Allan standard deviation curve and the black Allan standard deviation curve still maintain a high degree of consistency in trends at different time scales, and the −1/2 slope characteristic consistent with white noise in the red curve basically disappears, indicating that the “AR (p) + KF-A” method has a very effective suppression effect on the IFOG white noise. However, the green Allan standard deviation curve can be regarded as the result of the overall downward shift of the blue Allan standard deviation curve, and all slope characteristics in the blue curve are retained in the green curve, indicating that the suppression degree of the “ARMA (p, q) + Kalman” method on the IFOG noise is limited.

**Figure 5:**
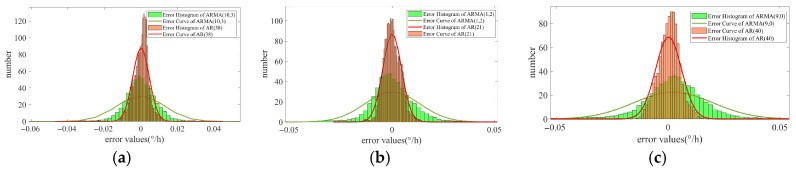
The fitting residual distributions of the two models for three groups of IFOG simulation data. (**a**) The first group; (**b**) the second group; (**c**) the third group.

The previous visual and qualitative analysis of the performance of the two IFOG random error modeling methods was conducted through Figure 3 and Figure 4. Next, the fitting accuracy of the two types of models to the IFOG output signals is quantitatively analyzed. The errors between the filtering results of the two methods and the time-correlated term simulation data are calculated, respectively. The error distribution histograms are shown in Figure 5, and the error analysis results are presented in Table 4.

It can be seen from Figure 5a–c that, compared with the ARMA modeling method based on the AIC criterion, the pure AR modeling method proposed in this paper has a narrower error distribution interval. Meanwhile, the analysis of the data in Table 4 shows that compared with the reference modeling method, the mean errors (MEs) between the predicted values of the modeling method proposed in this paper and the simulation data is overall reduced by an order of magnitude, indicating that the latter has better mean tracking performance. In the three groups of simulation experiments, the root mean square errors (RMSEs) of the latter are reduced by 60%, 68%, and 68%, respectively, compared with the former, further demonstrating that the modeling method proposed in this paper has better fitting accuracy.

Based on the above simulation results, it can be concluded that the ARMA model based on the AIC criterion only achieves overall fitting of the IFOG simulation data but fails to accurately express the single noise components in the data. This leads to incomplete suppression of each type of noise by the Kalman filter based on this model, and such fundamental limitations make it difficult to further improve the Kalman filtering effect. In contrast, the modeling method proposed in this paper can perform targeted model-order determination according to the data composition characteristics of the IFOG. Therefore, the pure AR model based on this method can accurately represent the time-correlated term information of the IFOG output across different time scales. The filtering results also show that the proposed method can break through the fundamental limitations of the reference method, achieving more thorough suppression of the white noise in IFOG data.

**Figure 6:**
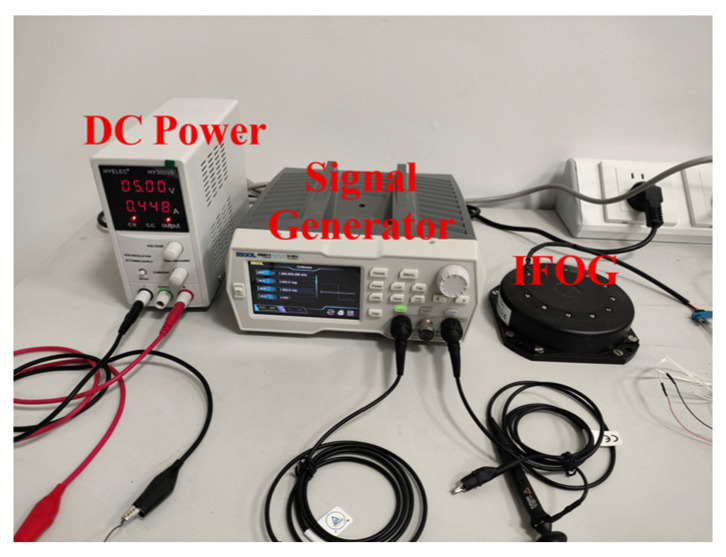
“120-type IFOG” and its supporting equipment.

It can be seen from the previous simulation experiments that the “AR (p) + KF-A” method proposed in this paper can break through the fundamental limitations of the “ARMA (p, q) + Kalman” method and more thoroughly suppress the white noise of IFOG, and it has stronger error separation ability. This section will further verify the effectiveness of the “AR (p) + KF-A” method based on three groups of IFOG measured data with a duration of 8 h (the three IFOGs are denoted as IFOG-A, IFOG-B, and IFOG-C, respectively), and use the “KF-B” filter mentioned in the paper to suppress the colored noise of the IFOG data. Select the first 10 min of each group of IFOG data as model training data, and the model information obtained from training is shown in Table 5. Figure 6 shows the “120-type IFOG” and its supporting equipment used in the experiment.

**Figure 7:**
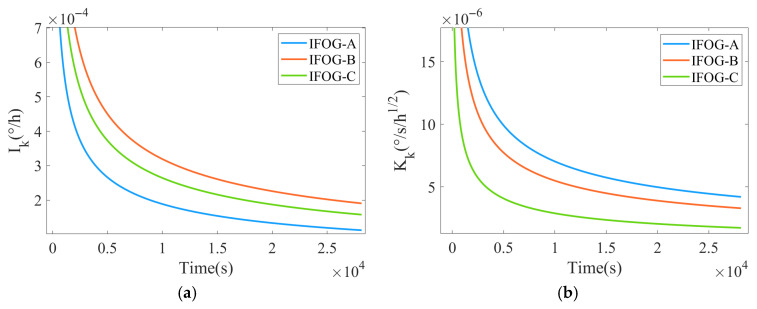
Real-time decoupling estimation results of colored noise coefficients. (**a**) BI noise PSD coefficient; (**b**) RRW noise PSD coefficient.

Substitute the AR models in Table 5 into the KF-A filter to suppress the white noise of IFOG. Meanwhile, the PSD coefficients of two types of colored noises, namely BI and RRW, are estimated in real time, and the coefficient estimation results are shown in Figure 7. It can be seen from Figure 7a,b that the noise coefficient estimation results of the three IFOGs all converge, which demonstrates the effectiveness of the real-time decoupling estimation method for colored noise coefficients proposed in this paper.

**Figure 8:**
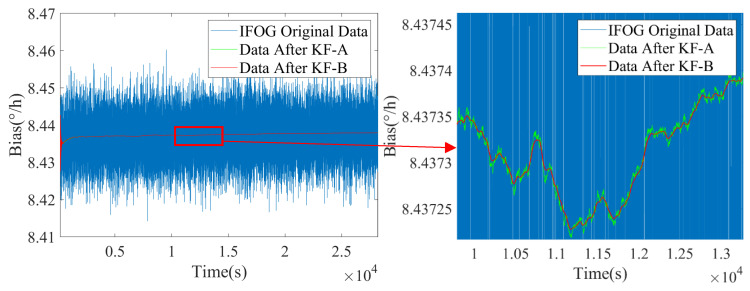
Noise suppression results of IFOG-A.

**Figure 9:**
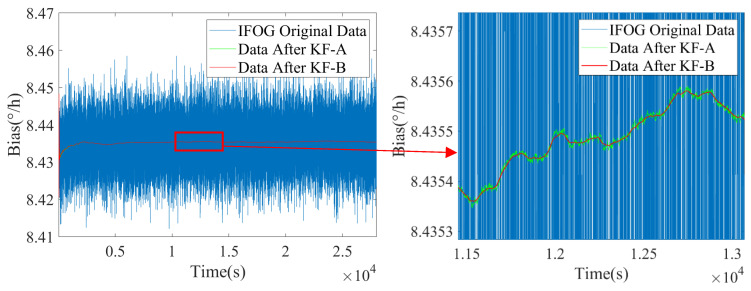
Noise suppression results of IFOG-B.

**Figure 10:**
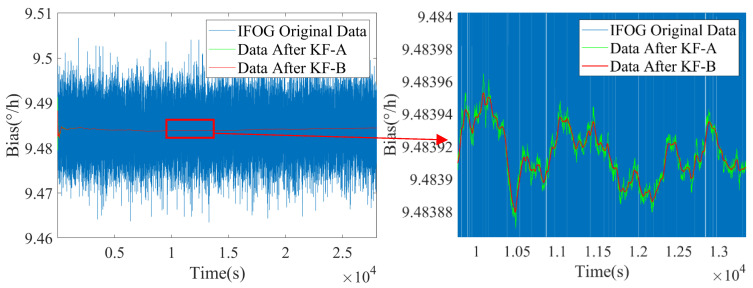
Noise suppression results of IFOG-C.

Based on the above real-time estimated values of noise coefficients ${\hat{I}}_{k}$ and ${\hat{K}}_{k}$, the state transition matrix of the KF-B filter is updated to further suppress the colored noise of IFOG. The filtering results are shown in Figure 8, Figure 9 and Figure 10, where the blue line represents the original IFOG data, the green line represents the IFOG data filtered by KF-A, and the red line represents the IFOG data filtered by KF-B. It can be seen from Figure 8, Figure 9 and Figure 10 that compared with the green lines, the high-frequency components in the red lines are significantly reduced, and the red lines maintain a high degree of consistency with the low-frequency trends of the green lines. This indicates that the KF-B filter can further and effectively suppress the high-frequency colored noise of IFOG on the basis of the KF-A filter.

**Figure 11:**
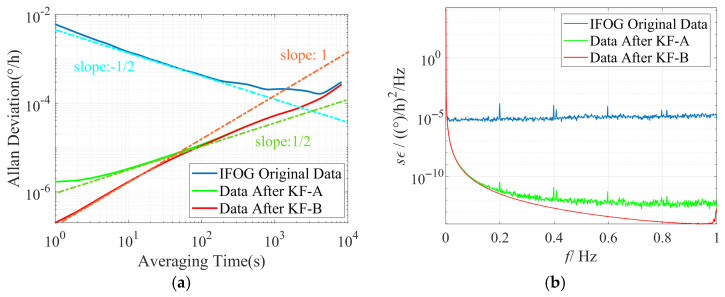
Time-domain and frequency-domain characteristics of IFOG-A before and after noise suppression. (**a**) Allan standard deviation curve; (**b**) PSD curve.

**Figure 12:**
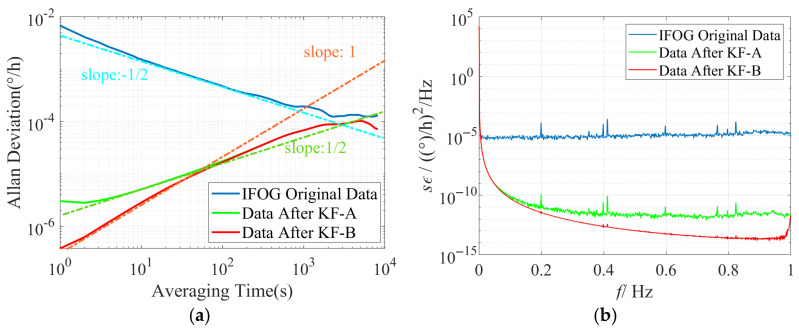
Time-domain and frequency-domain characteristics of IFOG-B before and after noise suppression. (**a**) Allan standard deviation curve; (**b**) PSD curve.

**Figure 13:**
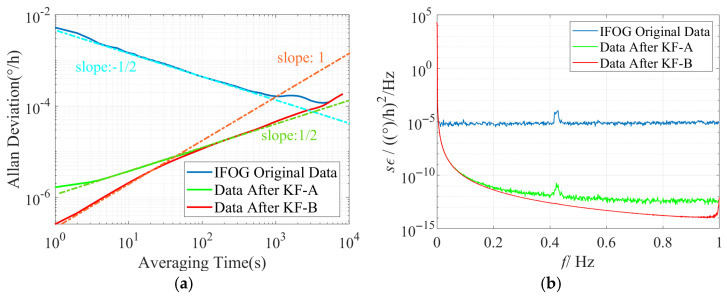
Time-domain and frequency-domain characteristics of IFOG-C before and after noise suppression. (**a**) Allan standard deviation curve; (**b**) PSD curve.

Conduct time-domain and frequency-domain analyses on the IFOG data before and after filtering, and plot the corresponding Allan standard deviation double-logarithmic curves and power spectral density curves for each group of experiments based on the above filtering results, as shown in Figure 11, Figure 12 and Figure 13. It can be seen from Figure 11a, Figure 12a and Figure 13a that the slope of the Allan standard deviation curve of the original data of the three IFOGs after KF-A filtering changes from −1/2 (consistent with the ARW error characteristic) to 1/2 (consistent with the RRW error characteristic), indicating that the KF-A filter effectively suppresses the ARW error. Further, the slope of the Allan standard deviation curve of the three IFOGs after KF-B filtering in the medium and high-frequency bands changes from the original 1/2 (consistent with the RRW error characteristic) to 1 (consistent with the RR error characteristic), which shows that the KF-B filter effectively suppresses the RRW error in the IFOG data. Among them, the remaining RR error after filtering is caused by the response distortion of the IFOG system to the linearly changing rate input. It has low-frequency characteristics and essentially belongs to the deterministic error of the system, which needs to be further compensated through dynamic modeling (such as polynomial fitting, neural network fitting, etc.). Since this error cannot be suppressed by filtering, it is not within the scope of this paper. Meanwhile, when Figure 11b, Figure 12b, and Figure 13b are observed, it can be seen that the original IFOG data is mainly composed of ARW error, and its corresponding PSD approximately exhibits a constant distribution (blue curve). The PSD curve of the data after KF-A filtering (green curve) shows typical power-law noise characteristics, with the power density in the medium and high-frequency bands reduced by more than 5 orders of magnitude compared with the original data. The PSD curve of the data after KF-B filtering (red curve) further reduces the medium and high-frequency power density by 1–2 orders of magnitude on the basis of the green curve. The above simulation and experimental results demonstrate the accuracy of the proposed method in modeling various noises in IFOG data and the effectiveness of noise suppression.

**Figure 14:**
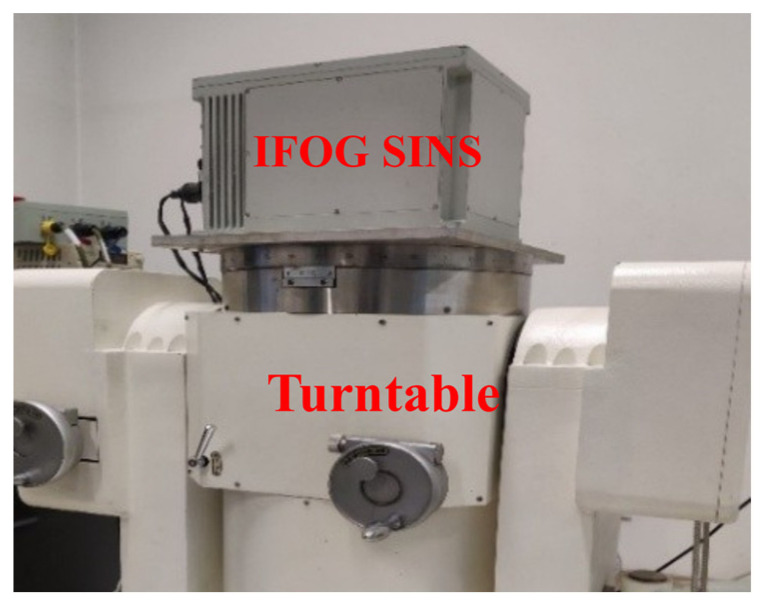
IFOG strapdown inertial navigation system and biaxial turntable.

**Figure 15:**
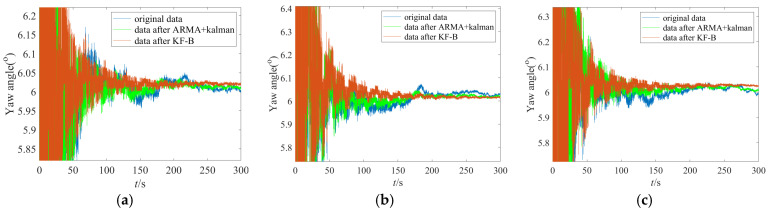
Yaw angle results of the first group of initial alignment tests. (**a**) The first data collection; (**b**) the second data collection; (**c**) the third data collection.

**Figure 16:**
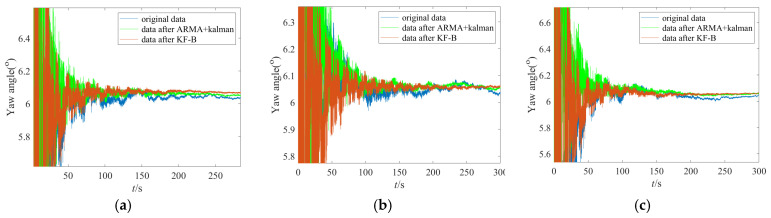
Yaw angle results of the second group of initial alignment tests. (**a**) The first data collection; (**b**) the second data collection; (**c**) the third data collection.

To more fully verify the effectiveness and advancement of the proposed IFOG random error suppression method in this paper, a static base initial alignment test of the IFOG strapdown inertial navigation system is carried out. Figure 14 shows the single-axis IFOG strapdown inertial navigation system used in the initial alignment test, with a data output frequency of 100 Hz. The bias stability of the IFOG is 0.012°/h, and the bias stabilities of the accelerometers are 58 μg, 49 μg, and 50 μg, respectively. The IFOG strapdown inertial navigation system is placed statically on a leveling turntable for data collection. A total of two groups of initial alignment tests were conducted. Each group involved three data collection operations, and experimental data with a duration of 5 min were collected each time for calculation. The equipment was powered on and off before the start of each group of tests. Since the alignment accuracy of horizontal attitudes (pitch angle, roll angle) depends on the accuracy of accelerometers, while the alignment accuracy of azimuth attitude (yaw angle) depends on the accuracy of gyroscopes, this section only analyzes the initial alignment accuracy of the yaw angle. The initial alignment results of the yaw angles for the two groups of tests are shown in Figure 15 and Figure 16. In the figures, the blue lines represent the alignment results based on the original data, the green lines represent the alignment results filtered by the “ARMA + Kalman” method, and the red lines represent the alignment results filtered by the “KF-B” method. It can be seen from Figure 15 and Figure 16 that the red lines have the fastest convergence speed and the smallest volatility in the last 2 min.

Further, a quantitative analysis is conducted on the initial alignment results in Figure 15 and Figure 16. The calculation results of the alignment accuracy (standard deviation) based on different filtering methods in each group of tests are shown in Table 6, and the calculation results of the dispersion (standard deviation) of the alignment results within the last 2 min of a single test are shown in Table 7.

Analyzing the experimental results data in Table 6 shows the following: In the first group of initial alignment tests, the heading angle alignment accuracy based on the original IFOG data is 90.73″, that based on the data filtered by the “ARMA + Kalman” method is 57.65″, and that based on the data filtered by the “KF-B” method is 11.60″. The heading angle alignment accuracy of the proposed method in this paper is improved by 51% compared with the “ARMA + Kalman” method. In the second group of initial alignment tests, the heading angle alignment accuracy based on the original IFOG data is 33.50″, that based on the data filtered by the “ARMA + Kalman” method is 32.29″, and that based on the data filtered by the “KF-B” method is 17.21″. The heading angle alignment accuracy of the proposed method is improved by 45% compared with the “ARMA + Kalman” method.

Analyzing the experimental results data in Table 7 shows the following: In the first group of initial alignment tests, the average standard deviation of the heading angle alignment results based on the original IFOG data in the last 2 min is 37.68″, that based on the data filtered by the “ARMA + Kalman” method is 21.89″, and that based on the data filtered by the “KF-B” method is 15.29″. The heading angle alignment volatility of the proposed method in the last 2 min is reduced by 18% compared with the “ARMA + Kalman” method. In the second group of initial alignment tests, the average standard deviation of the heading angle alignment results based on the original IFOG data in the last 2 min is 37.09″, that based on the data filtered by the “ARMA + Kalman” method is 23.98″, and that based on the data filtered by the “KF-B” method is 17.59″. The heading angle alignment volatility of the proposed method in the last 2 min is reduced by 17% compared with the “ARMA + Kalman” method.

Based on the comprehensive analysis of the above experimental results, the method proposed in this paper can effectively improve the initial alignment accuracy and convergence speed of the fiber-optic strapdown inertial navigation system.

## 6. Conclusions

This paper has proposed a method for suppressing random errors in IFOG under static conditions. First, the noise composition in IFOG data was deeply discussed, and the fundamental reason why the ARMA model based on the AIC criterion limits the performance of Kalman filtering was analyzed in depth. Based on the above conclusions, a pure AR model modeling method constrained by noise variance ratio information was proposed. Then, based on the pure AR model and the linear system equations of the main noises in IFOG, a decoupling method for power spectral density parameters between BI noise and RRW noise was derived. Finally, based on all the information obtained earlier, two adaptive Kalman filters based on physical information decoupling were designed, and they effectively suppress ARW noise and RRW noise, respectively, through progressive filtering. Both simulation results and two types of experimental results show that the method proposed in this paper achieves better effectiveness and advancement compared with traditional methods. This method uniquely decouples BI/RRW spectral parameters via hybrid AR-physical modeling, enabling >48% alignment accuracy gains.

The relevant theories proposed in this paper can provide an idea for the research on random error suppression of IFOG under static conditions, thereby further improving the measurement accuracy of IFOG at the algorithm compensation level, which is conducive to reducing the manufacturing cost of equipment such as IFOG strapdown inertial navigation. However, there are still some problems to be solved. For example, more complex noises will be introduced in dynamic environments (mainly including high-frequency vibration and low-frequency maneuvering), which increases the difficulty of identifying the prior information of noises. At the same time, the method proposed in this paper does not consider compensating for the remaining low-frequency noises after filtering, which will cause large errors in the long-term navigation process. Therefore, on the basis of the research conclusions of this paper, it is necessary to further carry out targeted research on the above existing problems.

## Figures and Tables

**Table 1:** Summary of the advantages of the new method compared with the traditional method.

Comparison Dimension	Traditional Rule-Based ARMA Modeling Method	New Method Proposed in This Study
Random Error Modeling	Has defects of manual intervention in order determination and poor modeling accuracy, and it lacks a standardized model form.	Establishes a standardized model form, proposes a new model-order criterion, achieves high-precision modeling of random errors, and avoids the problems of manual intervention and insufficient accuracy.
Colored Noise Processing	In colored noise scenarios, there are issues with the insufficient accuracy of the state equation and dimension expansion; unable to solve the coupling problem of main-colored noises such as angular-rate random walk and bias instability.	Combines the interpretability of linear system equations for each noise term with the fitting advantages of the ARMA model, derives a recursive estimation method for colored noise power spectral information, realizes the decoupling of main-colored noises, and accurately estimates their spectral parameters, and the method has strong interpretability.
Filter Design and Effect	Limited due to principled constraints such as insufficient modeling accuracy and difficulties in state vector dimension expansion, and it is difficult to effectively suppress noise terms that affect FOG measurement accuracy.	Designs a filter based on the decoupling of colored noise spectral information, dynamically suppresses white noise and colored noise, breaks through the principled limitations of traditional methods, and realizes the effective suppression of main angular-rate white noise terms and colored noise terms.

**Table 2:** Relevant parameter settings for IFOG simulation data.

Group	Gaussian White Noise Variance	First-Order Markov-Driven White Noise Variance	Anti-Time Correlation Coefficient
1	0.0030	1.5 × 10^−9^	0.9990
2	0.0060	1.1 × 10^−9^	0.9995
3	0.0090	8.9 × 10^−10^	0.9997

**Table 3:** Information of two random error models.

Group	AIC	ARMA (p, q)	η	AR (p)
1	−53,694.1	(10, 3)	0.9948	(38)
2	−40,973.7	(1, 2)	0.9971	(21)
3	−33,969.3	(9, 0)	0.9943	(40)

**Table 4:** Fitting mean errors and root mean square errors of the two models (°/h).

Group	ME _(ARMA)_	RMSE _(ARMA)_	ME _(AR)_	RMSE _(AR)_
1	1.2 × 10^−3^	0.012	8.6 × 10^−5^	0.005
2	1.2 × 10^−4^	0.015	5.9 × 10^−5^	0.005
3	2.4 × 10^−3^	0.018	3.3 × 10^−4^	0.006

**Table 5:** Model information of three IFOG datasets.

Group	Model	η
IFOG-A	AR (79)	0.9986
IFOG-B	AR (35)	0.9928
IFOG-C	AR (21)	0.9907

**Table 6:** Alignment accuracy of different filtering methods (°).

The First Group				The Second Group			
Collection	Original Data	ARMA + Kalman	After KF-B	Collection	Original Data	ARMA + Kalman	After KF-B
1	6.017	6.023	6.018	1	6.018	6.055	6.054
2	6.051	6.042	6.024	2	6.037	6.073	6.063
3	6.002	6.010	6.021	3	6.031	6.063	6.056
σ	0.025	0.016	0.003	σ	0.009	0.009	0.005

**Table 7:** Dispersion of alignment results within the last 2 min of a single test for different filtering methods (°).

The First Group				The Second Group			
Collection	Original Data	ARMA + Kalman	After KF-B	Collection	Original Data	ARMA + Kalman	After KF-B
1	0.011	0.006	0.004	1	0.009	0.007	0.006
2	0.010	0.005	0.005	2	0.013	0.007	0.004
3	0.010	0.007	0.004	3	0.009	0.006	0.005

## Data Availability

The original contributions presented in this study are included in the article.
